# Knowledge attitudes and practices towards long-acting antiretroviral therapy in HIV/AIDS patients

**DOI:** 10.1038/s41598-026-44035-0

**Published:** 2026-03-17

**Authors:** Meixin Ren, Junyi Duan, Tao Huang, Guanghui Zhang, Han Jia, Lin Jia, Yuan Fang, Luyao Zheng, Taiyi Jiang, Wen Wang, Caiping Guo, Tong Zhang

**Affiliations:** 1 Department of Infectious Diseases and Medical Immunology, Beijing Youan Hospital, Capital Medical University, Beijing, 100069 China; 2 Tian Yuan Studio, Beijing Youan Hospital, Capital Medical University, Beijing, 100069 China

**Keywords:** HIV infections, Antiretroviral therapy, Long-acting, Knowledge, Attitudes, Practice, Cross-sectional studies, Structural equation modeling, Diseases, Health care, Medical research

## Abstract

This study aimed to evaluate the knowledge, attitudes, and practices (KAP) regarding long-acting antiretroviral therapy (LA-ART) among HIV/AIDS patients. A cross-sectional study was conducted at the Infection Center of Beijing Youan Hospital between December 2024 and May 2025. Data were collected using a structured questionnaire comprising demographic variables and KAP-related items. A total of 919 questionnaires were collected; after excluding 93 responses (declined consent, abnormal height/weight entries, failed logic-check questions, and uniform responses), 826 valid questionnaires were included in the final analysis, yielding a valid response rate of 89.88%. Most participants resided in urban areas (62.0%) and were currently employed (72.0%), with over half (52.9%) holding an associate or bachelor’s degree. Their knowledge, attitude, and practice scores were 14.38 ± 5.52 (possible range: 0–36), 39.71 ± 4.36 (possible range: 12–60), and 22.29 ± 2.99 (possible range: 9–27), respectively. The structural equation modeling results showed that knowledge had direct effects on attitude (β = 0.427, *P* = 0.016) and practice (β = 0.132, *P* = 0.003). Meanwhile, attitude had a direct impact on practice (β = 0.460, *P* = 0.034). Furthermore, knowledge indirectly affected practice through attitude (β = 0.197, *P* = 0.014). HIV/AIDS patients demonstrated insufficient knowledge, a generally neutral attitude, yet reported positive practices regarding LA-ART. Future clinical interventions should prioritize structured, evidence-based educational programs to enhance patient understanding and shift attitudes.

**Supplementary Information:**

The online version contains supplementary material available at 10.1038/s41598-026-44035-0.

## Introduction

Human immunodeficiency virus (HIV) infection continues to represent one of the most formidable global health challenges of our time. By the end of 2024, approximately 40.8 million individuals worldwide were living with HIV, accompanied by an estimated 1.3 million new infections and 630,000 acquired immunodeficiency syndrome (AIDS)-related deaths within that year alone^1^. Although substantial advances have been achieved in reducing mortality rates and enhancing quality of life through expanded access to antiretroviral therapy (ART), HIV continues to exert a considerable disease burden characterized by lifelong treatment obligations, associated comorbidities, and persistent social stigma^2,3^. Within China specifically, despite a relatively low general-population prevalence (estimated at 0.058% at the end of 2011), more than one million individuals were living with HIV/AIDS by late 2020, with tens of thousands of new diagnoses being reported annually^4,5^. This persistent burden emphasizes the critical necessity for enhanced HIV management strategies that integrate evidence-based clinical care with comprehensive public health approaches^6^.

Currently, daily oral ART constitutes the established standard of care for HIV management, demonstrating remarkable efficacy in suppressing viral replication while simultaneously restoring immune function^7^. Nevertheless, maintaining adherence to daily dosing regimens can be significantly compromised by multiple factors, including pill fatigue, treatment-related adverse effects, and various logistical challenges, barriers that have been documented to influence regimen modifications in studies examining long-acting ART options^8^. A meta-analysis of adult studies reported that the mean proportion achieving optimal ART adherence (most commonly defined as ≥ 95%) was about 63%, with substantial variability across settings. These real-world gaps in adherence help explain why alternatives that reduce daily dosing demands have attracted increasing attention^9^. These adherence obstacles are further exacerbated by treatment burden and regimen fatigue, particularly in clinical contexts involving comorbidities, polypharmacy, and restricted medication access, as has been extensively reported across other chronic disease management settings^10^. Importantly, many individuals living with HIV perceive the requirement for daily pill-taking as both a psychological and logistical burden, thereby underscoring the potential value of alternative therapeutic approaches that can maintain viral suppression while simultaneously reducing dosing frequency^11^.

In response to these challenges, long-acting injectable ART (LA-ART) utilizing cabotegravir plus rilpivirine has emerged as an effective and generally well-tolerated alternative to conventional daily oral therapy for virologically suppressed individuals living with HIV^12,13^. However, broader implementation of LA-ART may be constrained by injection-site adverse events, visit logistics, and affordability. In the pooled Week 48 analysis of the Phase 3 ATLAS and FLAIR trials, 83% of participants receiving CAB plus RPV long-acting therapy reported injection-site reactions, and 1% discontinued treatment due to these events. In addition, the drug acquisition cost remains substantial; for example, Spanish list-price data reported a net monthly cost of 803.14 euros for CAB plus RPV long-acting therapy. These real-world tolerability and cost considerations may directly influence patient acceptance, especially in settings where access requires regular clinic attendance^14–16^.

Given these implementation challenges, understanding patients’ knowledge, attitudes, and practices (KAP) toward LA-ART becomes essential for effectively addressing existing barriers to adoption. The KAP framework represents a well-established public health methodology for systematically evaluating what individuals understand, how they perceive, and how they respond to specific health interventions^17,18^. Such comprehensive surveys possess the capability to identify critical misconceptions, implementation barriers, and motivational factors that significantly influence treatment decision-making processes, thereby providing valuable guidance for developing targeted educational initiatives and tailored intervention strategies^19^. While KAP studies have been extensively applied across various domains of HIV prevention and treatment research, relatively few investigations have specifically examined patient perceptions regarding LA-ART, and even fewer have concentrated on Chinese populations living with HIV^20–23^. In pivotal trials, 83% of participants receiving long-acting cabotegravir plus rilpivirine reported injection-site reactions, and the reported monthly cost reached €803.14 in Spain, highlighting practical barriers to implementation^14–16^. These challenges are particularly relevant in China, where over one million individuals are living with HIV^4,5^.

This context reveals a clear research gap: although qualitative studies have explored patient interest and perceived barriers to LA-ART^24,25^, there remains a lack of large-scale quantitative evidence on the KAP of Chinese patients toward this emerging treatment modality. More importantly, what remains unknown is the mechanism through which knowledge translates into behavioral intentions, an issue that descriptive surveys alone cannot adequately address.

Our specific contribution is to provide the first comprehensive KAP assessment of LA-ART in a major Chinese urban center using structural equation modeling (SEM) to test a hypothesized pathway in which knowledge influences practice both directly and indirectly through attitude. By offering a testable quantitative framework, this study aims to inform targeted educational strategies and policy planning. Therefore, this study aimed to evaluate the KAP regarding LA-ART among HIV/AIDS patients and to elucidate the interrelationships among these three domains.

## Methods

### Study design and participants

This cross-sectional study was conducted at the Infection Center of Beijing Youan Hospital between December 2024 and May 2025, targeting living with HIV/AIDS who met the eligibility criteria. Eligible participants were required to meet the following inclusion criteria: (1) a confirmed diagnosis of HIV infection from a qualified medical institution (e.g., hospital or Center for Disease Control and Prevention); (2) currently receiving standard daily oral antiretroviral therapy (ART); (3) a aged ≥ 18 years; (4) sufficient cognitive and communicative abilities to comprehend the questionnaire independently or with assistance from trained researchers. Participants were excluded from the study if they met any of the following conditions: (1) significant hearing, visual, or speech impairments that could not be effectively addressed with assistive measures (e.g., hearing aids, large-print questionnaires, sign language interpretation); or (2) any acute or serious medical condition that, in the investigators’ judgment, could interfere with understanding or completing the questionnaire. Ethical approval was obtained from the Beijing Youan Hospital Research Ethics Committee (LL-2024-150-K), and all participants provided written informed consent before being enrolled in the study.

### Questionnaire introduction and quality control

The questionnaire was developed with reference to established national and international guidelines relevant to HIV care and behavioral research^26^. The initial draft underwent revision based on expert feedback from 3 specialists in infectious diseases, epidemiology, and health behavior, ensuring content relevance and clarity. A pilot test was conducted with 67 participants, all of whom completed the questionnaire without missing data and met the established criteria for validity. The pilot participants were recruited only to refine the questionnaire and were not included in the final analysis. Logic-check questions were embedded in the final questionnaire to identify inconsistent or inattentive responses. The overall internal consistency of the instrument was excellent, as evidenced by a Cronbach’s α coefficient of 0.921.

The final version of the questionnaire consisted of 50 items spanning four dimensions: basic demographic and clinical information (18 items), knowledge (12 items), attitudes (11 items), and practices (9 items). The knowledge section applied a 3-point scale, assigning 2 points for “very familiar,” 1 point for “heard of it,” and 0 points for “unaware,” with a total possible score ranging from 0 to 36. The attitude section employed a 5-point Likert scale, ranging from 1 (“very negative”) to 5 (“very positive”). Items 2 and 5 through 11 were scored positively (i.e., a = 5 to e = 1), whereas items 1, 3, and 4 were reverse-coded (i.e., a = 1 to e = 5), resulting in a total score range of 12 to 60. The practice section uniformly scored all nine items on a 3-point frequency scale, with a = 3 (“frequently”), b = 2 (“occasionally”), and c = 1 (“never”), yielding a total score between 9 and 27. The final questionnaire was administered in Chinese. For reproducibility, the original Chinese questionnaire and the English translation are provided as Appendix 1 and 2. For interpretive purposes, knowledge scores were categorized as inadequate (0–18) or adequate (19–36); attitude scores as negative (12–30), neutral (31–42), or positive (43–60); and practice scores as negative (9–13), moderate (14–18), or positive (19–27).

To ensure appropriate participant selection, inclusion and exclusion criteria were strictly applied. The survey was conducted entirely online. A unique access code ensured that each participant could submit the questionnaire only once. Participants were invited through short message service (SMS) or WeChat, and instructed to complete the questionnaire on-site or at home in a quiet and private environment. They were advised that the estimated completion time was 15–25 min and were encouraged to consult trained staff if clarification was needed. However, investigators were trained to avoid leading or suggestive explanations. To ensure data validity, several quality control measures were implemented: all questions were mandatory, and one logic-check question was embedded to identify inattentive or inconsistent responses. No personally identifiable information (e.g., name, ID number, address, or phone number) was collected. Data were stored in password-protected electronic systems accessible only to authorized research staff, all of whom signed confidentiality agreements.

### Statistical analysis

#### Sample size

Sample size was estimated a priori using the standard formula for cross-sectional studies: n = Z²p(1 − p)/d². We used Z = 1.96 for a 95% confidence level, assumed *p* = 0.5 to maximize the required sample size, and set the admissible error at d = 0.05. The resulting theoretical minimum sample size was 480 after allowing an additional 20% for potential loss/exclusion. In the present study, 919 questionnaires were collected and 826 valid questionnaires were included in the final analysis, exceeding the estimated minimum.

Data analysis was performed using STATA version 17.0 (StataCorp, College Station, TX, USA). Continuous variables were expressed as mean ± standard deviation (SD), whereas categorical variables were summarized as frequencies and percentages (n, %). For normally distributed data, independent-samples t-tests were used to compare two groups, and one-way analysis of variance (ANOVA) was applied for comparisons involving three or more groups with homogeneity of variance. For non-normally distributed variables, the Mann–Whitney U test was employed. Spearman’s rank correlation analysis was conducted to examine associations among knowledge, attitude, and practice scores. The practice dimension was dichotomized using 70% of the maximum possible score as the cutoff to define high versus low practice levels^27^. Variables with a p-value < 0.1 in univariate analysis or deemed clinically relevant were entered into the multivariate logistic regression model. SEM was employed to further explore the interrelationships among knowledge, attitude, and practice, as well as the influence of baseline characteristics on these pathways. The hypothesized model assumed that knowledge directly influences both attitude and practice, and that attitude in turn directly affects practice, with the potential for knowledge to indirectly affect practice through attitude. Model fit was evaluated using standard indices, including the root mean square error of approximation (RMSEA), incremental fit index (IFI), Tucker–Lewis index (TLI), and comparative fit index (CFI). Acceptable model fit was defined as RMSEA < 0.08, IFI > 0.90, TLI > 0.90, and CFI > 0.90. Two-sided p-values < 0.05 were considered statistically significant.

## Results

### Demographic information on participants

A total of 919 questionnaires were collected, the following samples were excluded: (1) 27 cases declined participation consent; (2) 48 cases had abnormal height/weight entries; (3) 6 cases failed logic-check questions; (4) 8 cases selected “Unaware” uniformly for all items in the knowledge assessment section; (5) 4 cases selected “Neutral” uniformly for all items in the attitude assessment section.

This study included 826 HIV/AIDS patients (98.7% male, mean age 39.28 ± 9.42 years), predominantly urban residents (62.0%), with associate/bachelor’s degrees (52.9%), and employed (72.0%). Their knowledge, attitude, and practice scores were 14.38 ± 5.52 (possible range: 0–36), 39.71 ± 4.36 (possible range: 12–60), and 22.29 ± 2.99 (possible range: 9–27), respectively. Most participants had monthly incomes exceeding ¥2000 (88.7%), were aware of long-acting therapy (84.6%), and were currently receiving treatment (99.2%). Key demographic characteristics and KAP scores are summarized in Table 1.

**Table 1:** Demographic characteristics and KAP scores.

Variables	*N* (%)	Knowledge, mean ± SD	*P*	Attitude, mean ± SD	*P*	Practice, mean ± SD	*P*
*N* = 826							
Total score		14.38 ± 5.52		39.71 ± 4.36		22.29 ± 2.99	
Gender			0.804		0.735		0.416
Male	815(98.67%)	14.37 ± 5.50		39.70 ± 4.35		22.27 ± 3.00	
Female	11(1.33%)	14.73 ± 6.90		40.73 ± 5.35		23.09 ± 2.02	
Age	39.28 ± 9.42						
Residence			< 0.001		< 0.001		0.019
Rural	235(28.45%)	12.45 ± 5.83		38.83 ± 4.27		21.97 ± 2.93	
Urban	512(61.99%)	15.35 ± 5.19		40.29 ± 4.25		22.48 ± 2.99	
Suburban	79(9.56%)	13.81 ± 5.11		38.61 ± 4.79		21.96 ± 3.12	
Education			< 0.001		< 0.001		< 0.001
Junior high school or below	151(18.28%)	11.18 ± 5.98		38.07 ± 4.57		21.19 ± 3.28	
High school/technical secondary school	166(20.10%)	13.49 ± 5.12		39.55 ± 3.98		21.93 ± 3.03	
Associate’s/bachelor’s degree	437(52.91%)	15.35 ± 5.12		40.21 ± 4.26		22.69 ± 2.85	
Master’s degree or above	72(8.72%)	17.24 ± 4.33		40.51 ± 4.58		22.96 ± 2.38	
Employment status			< 0.001		< 0.001		< 0.001
Employed	595(72.03%)	15.05 ± 5.33		40.19 ± 4.11		22.52 ± 2.91	
Unemployed	184(22.28%)	12.66 ± 5.63		38.47 ± 4.74		21.69 ± 3.12	
Monthly income per capita			< 0.001		< 0.001		< 0.001
≤ 2000	93(11.26%)	11.63 ± 5.73		37.81 ± 5.13		21.44 ± 3.28	
2000–5000	293(35.47%)	13.41 ± 5.53		39.34 ± 3.84		22.03 ± 2.98	
5000–10,000	247(29.90%)	14.94 ± 5.09		40.02 ± 4.40		22.45±0.93	
10,000–20,000	138(16.71%)	16.20 ± 4.95		40.79 ± 4.16		22.78 ± 2.93	
≥ 20,000	55(6.66%)	17.05 ± 5.38		40.80 ± 4.77		23.07 ± 2.52	
Marital status			< 0.001		< 0.001		0.032
Married	256(30.99%)	13.18 ± 5.24		38.84 ± 4.03		22.01 ± 2.94	
Single	112(13.56%)	14.91 ± 5.56		40.10 ± 4.45		22.41 ± 3.01	
Duration since HIV diagnosis			0.573		0.398		0.465
Less than 1 year	69(8.35%)	13.97 ± 5.99		40.06 ± 4.61		21.80 ± 3.74	
1–3 years	78(9.44%)	14.55 ± 5.63		40.37 ± 4.28		22.81 ± 2.67	
3–5 years	149(18.04%)	13.99 ± 5.15		39.80 ± 4.10		22.29 ± 2.99	
More than 5 years	530(64.16%)	14.52 ± 5.55		39.54 ± 4.42		22.27 ± 2.92	
Receiving treatment			0.542		0.336		0.162
Yes	819(99.15%)	14.39 ± 5.48		39.73 ± 4.34		22.32 ± 2.93	
No	7(0.85%)	12.43 ± 9.54		37.29 ± 6.32		18.71 ± 6.60	
Anyone around with HIV infection			< 0.001		< 0.001		0.066
Yes	467(56.54%)	15.24 ± 5.29		40.18 ± 4.05		22.48 ± 2.83	
No	359(43.46%)	13.26 ± 5.62		39.10 ± 4.67		22.03 ± 3.17	
Heard of long-acting HIV therapy			< 0.001		< 0.001		< 0.001
Yes	699(84.62%)	15.32 ± 5.16		40.17 ± 4.16		22.56 ± 2.83	
No	127(15.38%)	9.21 ± 4.48		37.20 ± 4.62		20.80 ± 3.39	
Hypertension			0.973		0.158		0.488
Yes	103(12.47%)	14.27 ± 5.20		39.24 ± 4.23		22.16 ± 2.83	
No	723(87.53%)	14.39 ± 5.57		39.78 ± 4.38		22.30 ± 3.01	
Diabetes			0.284		0.050		0.185
Yes	41(4.96%)	13.22 ± 5.33		38.78 ± 4.42		21.78 ± 2.73	
No	785(95.04%)	14.44 ± 5.52		39.76 ± 4.36		22.31 ± 3.00	
Smoke frequently			0.527		0.683		0.201
Yes	254(30.75%)	14.17 ± 6.05		39.75 ± 4.48		22.19 ± 2.74	
No	572(69.25%)	14.47 ± 5.27		39.69 ± 4.32		22.33 ± 3.10	
Drink alcohol frequently			0.120		0.099		0.420
Yes	124(15.01%)	13.69 ± 5.92		39.06 ± 4.63		22.19 ± 2.92	
No	702(84.99%)	14.50 ± 5.44		39.83 ± 4.31		22.30 ± 3.00	
The main reason for choosing long-acting therapy (multiple choice)							
Reduced dosing frequency, no need to think about taking medication often	723(87.53%)						
Fewer clinic visits	563(68.16%)						
Avoid side effects of oral medications	587(71.07%)						
Reduced risk of privacy exposure	561(67.92%)						
Avoid carrying large amounts of medication during business trips or travel	588(71.19%)						
Other	38(4.60%)						
The main reason for not choosing long-acting therapy (multiple choice)							
Fear of injections	129(15.62%)						
High cost, financial pressure	737(89.23%)						
Inconvenience of regular visits to medical institutions for injections	296(35.84%)						
Concerns about side effects	313(37.89%)						
Worries about the efficacy of long-acting therapy	334(40.44%)						
Difficulty in adjusting treatment (e.g., dosage changes or medication switches)	192(23.24%)						
Other	27(3.27%)						

### Knowledge, attitude, and practice

The distribution of knowledge dimensions showed that the three questions with the highest number of participants choosing the ‘Unaware’ option were ‘HIV treatment primarily relies on ART, which suppresses viral replication, restores immune function, reduces complications, and significantly prolongs life expectancy.’ (K3) with 65.38%, ‘AIDS is a severe immune system disease caused by the HIV. HIV attacks the immune system, particularly CD4 + T cells, which are critical for coordinating the body’s defense against pathogens.’ (K1) with 64.77%, and ‘HIV infection progresses through stages: Acute infection; Asymptomatic/chronic infection; AIDS (late stage), where the immune system is severely damaged, leading to symptoms like persistent fever, weight loss, chronic diarrhea, fatigue, and swollen lymph nodes, etc.’ (K2) with 62.35% (Table 2).

**Table 2:** Distribution of knowledge dimension responses.

	*N* (%)				
	Strongly agree	Agree	Neutral	Disagree	Strongly disagree
1. Currently, HIV cannot be cured and requires lifelong treatment, causing significant physical and psychological stress.	400(48.43%)	265(32.08%)	133(16.10%)	20(2.42%)	8(0.97%)
2. You believe it is important to learn about advancements in HIV treatment and research.	521(63.08%)	265(32.08%)	36(4.36%)	2(0.24%)	2(0.24%)
3. You often worry about missing doses of your HIV medication.	398(48.18%)	283(34.26%)	119(14.41%)	17(2.06%)	9(1.09%)
4. You find it inconvenient to carry medication or remember daily doses.	380(46.00%)	294(35.59%)	130(15.74%)	17(2.06%)	5(0.61%)
5. You are willing to try long-acting therapy for HIV treatment.	339(41.04%)	296(35.84%)	170(20.58%)	17(2.06%)	4(0.48%)
6. You believe long-acting therapy has significant advantages in HIV/AIDS treatment.	335(40.56%)	327(39.59%)	149(18.04%)	11(1.33%)	4(0.48%)
7. You would recommend long-acting therapy to other people living with HIV.	331(37.65%)	334(40.44)	156(18.89)	17(2.06%)	8(0.97%)
8. Compared to daily oral medication, you believe long-acting therapy reduces dosing frequency and improves quality of life.	385(46.61%)	342(41.40%)	84(10.17%)	12(1.45%)	3(0.36%)
9. Compared to daily oral medication, you believe long-acting therapy helps protect privacy, and you have a positive attitude toward this benefit.	399(48.31%)	336(40.68%)	83(10.05%)	5(0.61%)	3(0.36%)
10. Compared to daily oral medication, you believe long-acting therapy reduces psychological stress from missed doses.	398(48.18%)	340(41.16%)	78(9.44%)	7(0.85%)	3(0.36%)
11. Compared to daily oral medication, you believe long-acting therapy is more convenient (e.g., no need to carry medication while traveling).	413(50.00%)	337(40.80%)	69(8.35%)	3(0.36%)	4(0.48%)

Responses to the attitude dimension showed that only 37.65% strongly agreed that they would recommend long-acting therapy to other people living with HIV (A7), only 40.56% strongly agreed that they believe long-acting therapy has significant advantages in HIV/AIDS treatment (A6), and only 41.04% strongly agreed that they are willing to try long-acting therapy for HIV treatment (A5) (Table 3).

**Table 3:** Distribution of attitude dimension responses.

	*N* (%)				
	Strongly agree	Agree	Neutral	Disagree	Strongly disagree
1. Currently, HIV cannot be cured and requires lifelong treatment, causing significant physical and psychological stress.	400(48.43%)	265(32.08%)	133(16.10%)	20(2.42%)	8(0.97%)
2. You believe it is important to learn about advancements in HIV treatment and research.	521(63.08%)	265(32.08%)	36(4.36%)	2(0.24%)	2(0.24%)
3. You often worry about missing doses of your HIV medication.	398(48.18%)	283(34.26%)	119(14.41%)	17(2.06%)	9(1.09%)
4. You find it inconvenient to carry medication or remember daily doses.	380(46.00%)	294(35.59%)	130(15.74%)	17(2.06%)	5(0.61%)
5. You are willing to try long-acting therapy for HIV treatment.	339(41.04%)	296(35.84%)	170(20.58%)	17(2.06%)	4(0.48%)
6. You believe long-acting therapy has significant advantages in HIV/AIDS treatment.	335(40.56%)	327(39.59%)	149(18.04%)	11(1.33%)	4(0.48%)
7. You would recommend long-acting therapy to other people living with HIV.	331(37.65%)	334(40.44%)	156(18.89)	17(2.06%)	8(0.97%)
8. Compared to daily oral medication, you believe long-acting therapy reduces dosing frequency and improves quality of life.	385(46.61%)	342(41.40%)	84(10.17%)	12(1.45%)	3(0.36%)
9. Compared to daily oral medication, you believe long-acting therapy helps protect privacy, and you have a positive attitude toward this benefit.	399(48.31%)	336(40.68%)	83(10.05%)	5(0.61%)	3(0.36%)
10. Compared to daily oral medication, you believe long-acting therapy reduces psychological stress from missed doses.	398(48.18%)	340(41.16%)	78(9.44%)	7(0.85%)	3(0.36%)
11. Compared to daily oral medication, you believe long-acting therapy is more convenient (e.g., no need to carry medication while traveling).	413(50.00%)	337(40.80%)	69(8.35%)	3(0.36%)	4(0.48%)

Responses to the practice dimension showed that 22.03% never try to quit smoke when knowing the risks of smoking (P2), 17.80% never try to reduce/quit drink alcohol when knowing the risks of alcohol (P3). Meanwhile, even if the current regimen is inconvenient, 18.28% never considered switching treatment regimens (P5) (Table 4).

**Table 4:** Distribution of practice dimension responses.

	*N* (%)		
	Frequently	Occasionally	Never
1. How often do you actively learn about long-acting HIV therapy	230(27.85%)	534(64.65%)	62(7.51%)
2. If you smoke, how often do you try to quit, knowing the risks of smoking	371(44.92%)	273(33.05%)	182(22.03%)
3. If you drink alcohol, how often do you try to reduce/quit, knowing the risks of alcohol	389(47.09%)	290(35.11%)	147(17.80%)
4. Do you hide or disguise your HIV medication to avoid others knowing your status	705(85.35%)	87(10.53%)	34(4.12%)
5. Would you consider switching treatments due to inconvenience with your current regimen	242(29.30%)	433(52.42%)	151(18.28%)
6. If on long-acting therapy, would you adhere to scheduled injections as prescribed	620(75.06%)	174(21.07%)	32(3.87%)
7. If on long-acting therapy, would you follow self-care guidelines for injection treatment	633(76.63%)	161(19.49%)	32(3.87%)
8. If on long-acting therapy, would you proactively undergo required tests (e.g., blood tests, liver/kidney function, CD4 count, viral load)	703(85.11%)	112(13.56%)	11(1.33%)
9. Do you have concerns about long-acting therapy due to its early-stage adoption in China (e.g., efficacy, side effects)	363(43.95%)	398(48.18%)	65(7.87%)

### Preliminary correlation analysis

As a preliminary assessment, knowledge, attitude, and practice scores were positively correlated (knowledge with attitude: *r* = 0.410, *P* < 0.001; knowledge with practice: *r* = 0.385, *P* < 0.001; attitude with practice: *r* = 0.315, *P* < 0.001) (Table 5). These bivariate correlations are provided for descriptive context, while the associations were further examined using multivariate logistic regression and structural equation modeling.

**Table 5:** Correlation analysis.

	Knowledge	Attitude	Practice
Knowledge	1		
Attitude	0.410 (*P* < 0.001)	1	
Practice	0.385 (*P* < 0.001)	0.315 (*P* < 0.001)	1

### Univariate and multivariate analysis for practice

Multivariate logistic regression showed that knowledge score (OR = 1.085, 95% CI: [1.048–1.123], *P* < 0.001), attitude score (OR = 1.104, 95% CI: [1.061–1.148], *P* < 0.001), and with associate’s/bachelor’s degree (OR = 1.870, 95% CI: [1.161–3.013], *P* = 0.010) were independently associated with proactive practice (Table 6).

**Table 6:** Univariate and multivariate analysis for practice dimension.

	Univariate logistic regression		Multivariate logistic regression	
	OR (95%CI)	*P*	OR (95%CI)	*P*
Knowledge score	1.135 (1.103–1.169)	< 0.001	1.085 (1.048–1.123)	< 0.001
Attitude score	1.157 (1.116-1.200)	< 0.001	1.104 (1.061–1.148)	< 0.001
Gender				
Male	1.023 (0.297–3.525)	0.971		
Female	ref			
Residence				
Rural	1.037 (0.621–1.733)	0.889	1.461(0.813–2.625)	0.204
Urban	1.766 (1.091–2.859)	0.021	1.291(0.761–2.190)	0.344
Suburban	ref		ref	
Education				
Junior high school or below	ref		ref	
High school/technical secondary school	1.587 (1.018–2.474)	0.042	1.219(0.739–2.012)	0.438
Associate’s/bachelor’s degree	2.921(1.994–4.278)	< 0.001	1.870(1.161–3.013)	0.010
Master’s degree or above	2.810(1.542–5.123)	< 0.001	1.401(0.673–2.915)	0.367
Employment status				
Employed	1.594(1.167–2.176)	0.003	0.977(0.668–1.430)	0.906
Unemployed	ref		ref	
Monthly income per capita				
≤ 2000	ref		ref	
2000–5000	1.314 (0.823–2.099)	0.253	0.948(0.548–1.638)	0.847
5000–10,000	2.031(1.248–3.306)	0.004	1.134(0.612–2.102)	0.689
10,000–20,000	2.656(1.522–4.634)	< 0.001	1.178(0.581–2.389)	0.650
≥ 20,000	2.500 (1.218–5.133)	0.013	1.138(0.469–2.763)	0.775
Marital status				
Married	0.708(0.522–0.959)	0.026	1.001(0.709–1.412)	0.995
Single	ref		ref	
Duration since HIV diagnosis				
Less than 1 year	0.784(0.471–1.304)	0.348		
1–3 years	1.278(0.766–2.134)	0.348		
3–5 years	1.092(0.745–1.599)	0.653		
More than 5 years	ref			
Receiving treatment				
Yes	2.406(0.535–10.825)	0.252		
No	ref			
Anyone around with HIV infection				
Yes	1.359(1.020–1.809)	0.036	0.969(0.697–1.347)	0.852
No	ref		ref	
Heard of long-acting HIV therapy				
Yes	2.671(1.818–3.924)	< 0.001	1.235(0.788–1.935)	0.357
No	ref		ref	
Hypertension				
Yes	0.787(0.516–1.199)	0.264		
No	ref			
Diabetes				
Yes	0.701(0.372–1.321)	0.271		
No	ref			
Smoke frequently				
Yes	0.976(0.718–1.328)	0.878		
No	ref			
Drink alcohol frequently				
Yes	0.798(0.540–1.180)	0.259		
No				

### SEM analysis

**Fig. 1:**
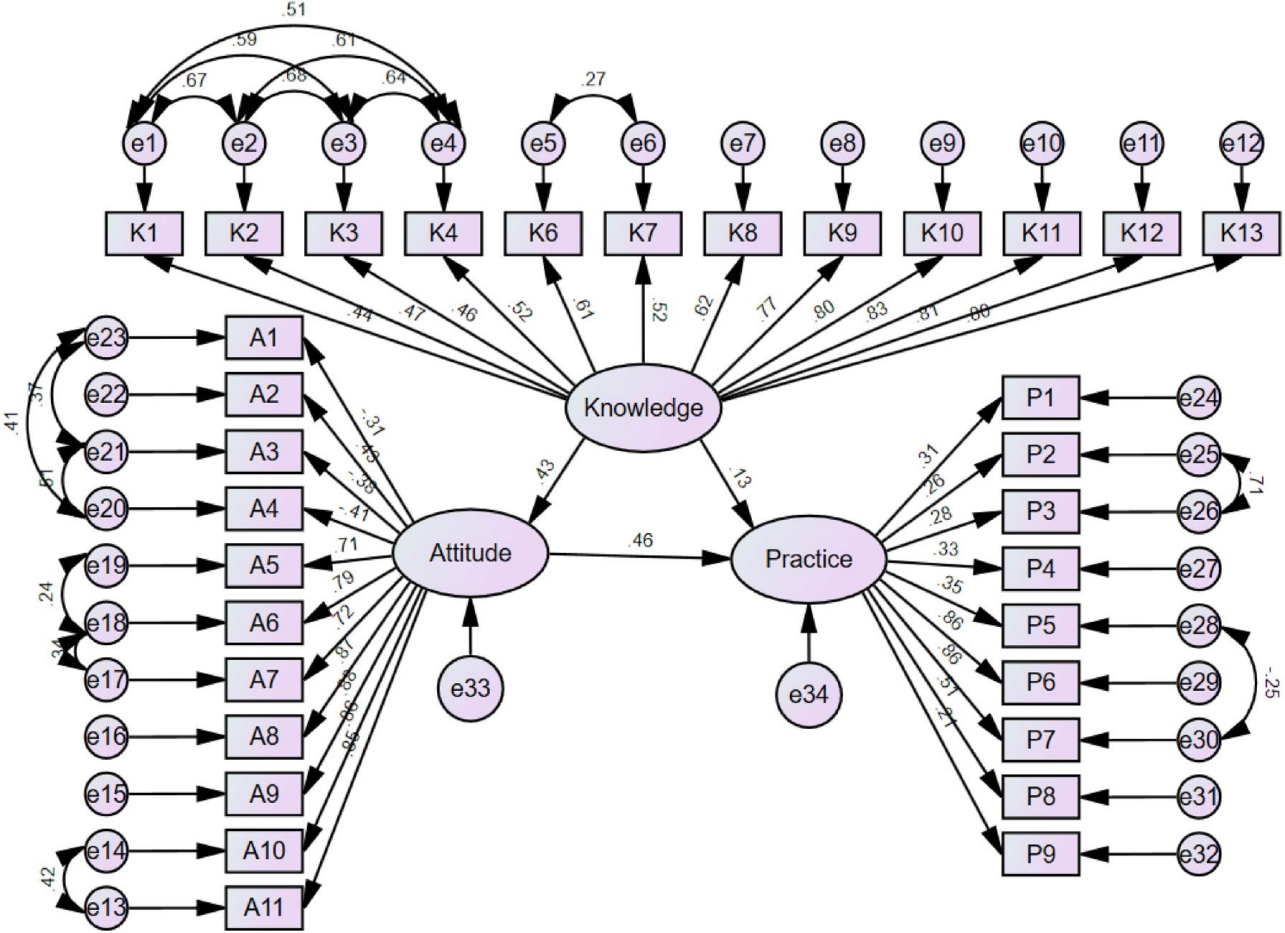
SEM model.

The fit of the SEM model yielded good indices demonstrating good model fit (CMIN/DF = 3.535; IFI = 0.923; TLI = 0.914; CFI = 0.923) (Table S1). The SEM results showed that knowledge had direct effects on attitude (β = 0.427, *P* = 0.016) and practice (β = 0.132, *P* = 0.003). Meanwhile, attitude had a direct impact on practice (β = 0.460, *P* = 0.034). Furthermore, knowledge indirectly affected practice through attitude (β = 0.197, *P* = 0.014) (Table S2 and Fig. 1).

## Discussion

HIV/AIDS patients exhibited insufficient knowledge and neutral attitudes, yet their reported practices were positive. This disconnect underscores that decision-making around LA-ART is shaped not only by knowledge, but also by treatment burden and pragmatic considerations.

Substantial gaps in fundamental HIV and ART knowledge were observed. These knowledge gaps were most pronounced for basic HIV and ART concepts (Table 2), indicating that long-term engagement in care does not necessarily translate into comprehensive understanding of disease mechanisms or emerging treatment modalities. These disparities align with evidence that health knowledge often reflects broader structural inequalities in education and information access^28^. Importantly, even among patients on ART, basic HIV education has not kept pace with the need to support informed decision-making regarding newer therapeutic options. Although knowledge gaps were evident, participants’ attitudes were not overtly negative. Instead, hesitancy appeared to stem from systemic concerns particularly affordability, long-term safety, and accessibility rather than lack of awareness alone. In our sample, cost was the most frequently cited barrier (89.23%), followed by concerns about efficacy (40.44%) and side effects (37.89%), and 35.84% reported the inconvenience of regular visits for injections (Table 1). In addition, 84.62% had heard of long-acting HIV therapy (Table 1), suggesting that reluctance was not solely driven by lack of awareness. This is consistent with prior studies highlighting structural and psychological barriers to LA-ART adoption^14,29,30^.

In contrast, participants showed willingness to adopt LA-ART. This inclination appears to be motivated less by detailed knowledge than by the desire to reduce daily treatment burden, manage stigma, and protect privacy. For many, the perceived convenience and discretion of LA-ART outweighed informational limitations, a pattern echoed in studies of other chronic conditions^31–33^. In this sample, these views primarily reflect working-age men (98.7% male; mean age 39.28 ± 9.42), most of whom were employed (72.0%) and lived in urban areas (62.0%) (Table 1). At the same time, privacy considerations were common: 67.92% selected reduced risk of privacy exposure as a reason to consider long-acting therapy (Table 1), and 85.35% reported frequently hiding or disguising HIV medication (Table 4). These characteristics suggest that LA-ART discussions and services in routine care need to address cost and safety questions directly, while offering visit arrangements that fit work schedules and reduce unwanted disclosure.

These findings reinforce the relevance of the KAP model while highlighting its contextual flexibility. Knowledge shaped perceptions, attitudes mediated decision-making, and practices were ultimately determined by a mix of informational, psychosocial, and systemic factors. This interpretation is supported by both correlation and SEM analyses, which showed that knowledge was positively associated with both attitude and practice, while attitude exerted a stronger direct effect on practice than knowledge alone. The indirect pathway from knowledge to practice via attitude further suggests that information may influence behavior primarily by shaping patients’ perceptions and readiness to act. In contexts where innovative therapies are not yet fully integrated into healthcare systems, structural barriers such as stigma, treatment fatigue, and service delivery models become especially influential^34^. Similar KAP patterns have been reported in studies of HIV and other chronic conditions, where knowledge alone was insufficient to ensure behavior change unless accompanied by favorable attitudes and feasible service structures^31,35^. Education showed a clear gradient across KAP scores in Table 1. Participants with higher educational attainment had higher knowledge and attitude scores and reported more proactive practices, which may translate into greater readiness to evaluate and adopt LA-ART when it becomes available in routine care. Social condition may also shape how knowledge translates into practice. In our sample, practice scores differed across income strata in Table 1, and cost was the most frequently reported reason for not choosing long-acting therapy (Table 1). This suggests that, even when patients are aware of LA-ART, socioeconomic constraints and related access factors may limit the ability to act on that knowledge, particularly when adoption requires regular clinic visits and additional out-of-pocket expenses. Therefore, the observed knowledge–practice association should be interpreted within this social context: improving knowledge alone may be insufficient if financial and service-delivery barriers remain, and patients with lower socioeconomic resources may require more structured counseling and support to achieve equitable uptake when LA-ART becomes more widely available.

In Table 6, proactive practice was associated with higher knowledge (adjusted OR = 1.085, 95% CI: 1.048–1.123; *P* < 0.001) and higher attitude scores (adjusted OR = 1.104, 95% CI: 1.061–1.148; *P* < 0.001). Education also remained associated with practice: compared with junior high school or below, having an associate’s/bachelor’s degree was linked to higher odds of proactive practice (adjusted OR = 1.870, 95% CI: 1.161–3.013; *P* = 0.010). This points to a practical gap for LA-ART rollout: patients with lower baseline knowledge or less education may need simpler, more structured counseling before they can act on interest in long-acting options. Several variables that matter for delivery (e.g., residence, prior awareness of long-acting therapy, income) were not retained as independent factors after adjustment, but they still help frame access and follow-up logistics.

These results hold several implications. First, educational strategies should not only increase awareness of LA-ART but also address patients’ real-world concerns, particularly cost, safety, and efficacy. Clinicians should engage patients in structured discussions to support informed decisions. Second, the strong patient interest in LA-ART, despite attitudinal hesitancy and limited knowledge, indicates an unmet need for simplified, stigma-reducing treatment options. Policy interventions such as inclusion of LA-ART in insurance coverage or cost-sharing subsidies may be critical for equitable access^30,36^. In China, standard oral ART is largely delivered through government-supported programs, but patients may still incur out-of-pocket expenses for related care and, in some settings, for newer regimens that are not yet routinely reimbursed. In this context, the high frequency of cost concerns in our sample likely reflects both direct affordability and anticipated expenses linked to injection-based follow-up (e.g., repeated visits and transport), as well as uncertainty about reimbursement.

### Limitations

Several limitations warrant consideration. This was a single-center, cross-sectional study with a predominantly male sample (98.7%), potentially limiting generalizability to women and rural populations. The reliance on self-reported data may introduce recall and social desirability biases, particularly in reporting positive practices. Future research could integrate quantitative detection methods to enhance the objectivity and accuracy of outcome measurements. Lastly, the study design does not allow for causal inference between knowledge, attitudes, and practices, although SEM was employed to explore relational pathways.

## Conclusion

HIV/AIDS patients in our study demonstrated insufficient knowledge and neutral attitudes but reported positive practices regarding LA-ART. These findings suggest that patients’ willingness to adopt long-acting regimens is driven more by treatment fatigue and lifestyle considerations than by factual understanding. Future strategies should integrate targeted education with system-level reforms to address financial and logistical barriers. At the patient level, targeted education is crucial to build knowledge and confidence. Concurrently, health system interventions must address financial and structural barriers to ensure equitable access. This comprehensive strategy is vital for the successful and sustainable adoption of long-acting therapies.

## Supplementary Information

Below is the link to the electronic supplementary material.

Supplementary Material 1

Supplementary Material 2

Supplementary Material 3

## Data Availability

All data generated or analysed during this study are included in this published article.
