# Supplementary material for: Knowledge attitudes and practices towards long-acting antiretroviral therapy in HIV/AIDS patients
Source: Sci Rep. 2026 Mar 17;16:15840. doi: 10.1038/s41598-026-44035-0 (PMC13195075; doi:10.1038/s41598-026-44035-0)
Supplement: Supplementary file 1 — Supplementary Material 1 [file 41598_2026_44035_MOESM1_ESM.docx]

**Table S1. SEM fit indicators**

| **Model 1** | **Ref.** | **Measured results** |
| --- | --- | --- |
| **CMIN/DF** | 1-3 excellent, 3-5 good | 3.535 |
| **IFI** | >0.8 good | 0.923 |
| **TLI** | >0.8 good | 0.914 |
| **CFI** | >0.8 good | 0.923 |

**Table S2. Analysis of direct and indirect effects**

| **Model paths** | **Total effects** | | **Standardized direct effects** | | **Standardized indirect effects** | |
| --- | --- | --- | --- | --- | --- | --- |
|  | **β (95%CI)** | **P** | **β (95%CI)** | **P** | **β (95%CI)** | **P** |
| Knowledge→Attitude | 0.427(0.355-0.492) | 0.016 | 0.427(0.355-0.492) | 0.016 |  |  |
| Knowledge→Practice | 0.328(0.260-0.418) | 0.003 | 0.132(0.052-0.228) | 0.003 | 0.197(0.151-0.257) | 0.014 |
| Attitude→Practice | 0.460(0.348-0.536) | 0.034 | 0.460(0.348-0.536) | 0.034 |  |  |
