# Supplementary material for: Knowledge attitudes and practices towards long-acting antiretroviral therapy in HIV/AIDS patients
Source: Sci Rep. 2026 Mar 17;16:15840. doi: 10.1038/s41598-026-44035-0 (PMC13195075; doi:10.1038/s41598-026-44035-0)
Supplement: Supplementary file 2 — Supplementary Material 2 [file 41598_2026_44035_MOESM2_ESM.docx]

我已知晓并同意将所收集的数据用于科学研究。

A 是，我同意参加 B 否，我不同意参加

第一部分 基本信息

1.您的性别:

a. 男

b. 女

2.您的年龄:________________

3.您的居住地:

a.农村

b.城市

c.城郊

4.您的教育程度:

a.初中及以下

b.高中/中专

c.大专/本科

d.硕士及以上

5.您的工作现状:

a.在职

b.待业

c.退休等其他情况

6.过去一年，您家每月人均收入为(包含实物收入和租房收入等):______元

a.&lt;2000

b.2000-5000

c.5000-10000

d.10000-20000

e.&gt;20000

7.您的婚姻状况:

a.已婚

b.未婚

c.离异、丧偶等其他情况

8.HIV确诊已经有多久时间:

a.1年以内

b.1-3年

c.3-5年

d.5年以上

9.您是否正在接受治疗:

a.是

b.否

10.您周围人是否有人有过HIV感染经历:

a.是

b.否

11.您的身高体重是:

a.身高_______________cm

b.体重_______________kg

12.您是否听说过长效艾滋病疗法:

a.是

b.否

13.您之前是否患过高血压:

a.是

b.否

14.您之前是否换过糖尿病:

a.是

b.否

15.您是否经常吸烟:

a.是

b.否

16.您是否经常饮酒:

a.是

b.否

17.如果您选择长效疗法，主要原因是:

a.减少给药频率，不用时常想着吃药

b.减少就诊次数

c.避免口服药物副作用

d.减少隐私暴露机会

e.避免出差、旅行等情况携带大量药物

f.其他（请补充）:

18.如果您不选择长效疗法，主要原因是:

a.因为害怕注射不选择长效疗法

b.因为费用较高，经济压力大

c.因为定期到医疗机构注射不方便

d.担心副作用

e.因为担心长效疗法的治疗效果

f.因为调整治疗不方便，比如需调整剂量或更换药物等

g.其他（请补充）:

第二部分 HIV/AIDS患者对于长效艾滋病疗法的知识

下列陈述，您了解的程度是:

1.艾滋病（AIDS）是由人类免疫缺陷病毒（HIV）引起的一种严重的免疫系统疾病;HIV病毒破坏人体的免疫系统，特别是CD4+ T细胞，这些细胞是免疫系统的重要组成部分，负责协调身体对病原体的防御反应。

a.很了解 b.听说过 c.不清楚

2.HIV感染可以分为急性感染期、无症状期（慢性感染期）和艾滋病期。艾滋病期是HIV感染的最晚期，此时免疫系统已严重受损;艾滋病患者可能会出现一系列症状，包括持续发热、体重减轻、慢性腹泻、疲劳、淋巴结肿大等。

a.很了解 b.听说过 c.不清楚

3.艾滋病的治疗主要是通过抗逆转录病毒治疗（ART），这种治疗可以抑制病毒复制，恢复免疫功能，减少并发症的风险，并显著延长患者的寿命。

a.很了解 b.听说过 c.不清楚

4.艾滋病功能性治愈是指在停用抗逆转录病毒治疗（ART）后，患者体内的HIV-1病毒载量仍然维持在检测下限，同时CD4+T淋巴细胞数量和机体免疫功能保持正常水平。

a.很了解 b.听说过 c.不清楚

5.中国的首都是上海。

a.是 b.否

6.可以通过联合使用多种抗病毒药物来抑制HIV的复制。药物种类包括核苷类反转录酶抑制剂（NRTIs）、非核苷类反转录酶抑制剂（NNRTIs）、蛋白酶抑制剂（PIs）、整合酶抑制剂（INSTIs）、融合抑制剂（FIs）及CCR5抑制剂等。

a.很了解 b.听说过 c.不清楚

7. 目前常用口服抗病毒药物可能出现的副作用:如恶心呕吐、腹泻，头痛头晕、抑郁、睡眠障碍等，贫血，皮疹，肝肾功能损伤、乳酸酸中毒、骨质疏松、血脂升高、体重增加等。

a.很了解 b.听说过 c.不清楚

8. 长效治疗药物的副作用:如注射部位疼痛、胃肠道不适、发热和乏力、肝功能异常、贫血、头痛、头晕和眩晕、脂肪代谢异常、血糖异常等。

a.很了解 b.听说过 c.不清楚

9.多数抗病毒药物为口服药物，需要每日服用;部分抗病毒药物为注射药物。目前长效疗法即以注射药物组合为主，无需每日应用。

a.很了解 b.听说过 c.不清楚

10. 我国批准的首个完整的HIV-1长效治疗方案:卡替拉韦注射液和利匹韦林注射液（最长每两个月注射一次）;卡替拉韦片剂也被批准与利匹韦林片剂联用，可选择作为注射疗法开始前口服导入用药，或作为未按计划进行注射治疗者的口服治疗方案。

a.很了解 b.听说过 c.不清楚

11. 长效治疗方案通常适用于已稳定控制病毒载量且对特定药物无耐药性的HIV患者。这意味着患者体内的HIV病毒载量已经降低到检测不到的水平或低于一定的阈值。

a.很了解 b.听说过 c.不清楚

12.长效艾滋病疗法的研究和临床试验正在积极推进，旨在减少用药频率，减少就诊频次，避免因漏服药物影响疗效，避免口服药物的副作用，提高治疗便利性，保护了患者的隐私。

a.很了解 b.听说过 c.不清楚

13.虽然长效治疗方案如卡替拉韦和利匹韦林的联合治疗可以将注射频率降低到每两个月一次，但仍然需要专业人员定期进行注射，这可能对一些患者来说仍然是一个负担;且仍存在一些副作用，如胃肠道不适;发热和乏力;肝功能异常等。

a.很了解 b.听说过 c.不清楚

第三部分 HIV/AIDS患者对于长效艾滋病疗法的态度

1.目前艾滋病无法治愈，需终身治疗，给您造成了巨大的身体与心理压力。(N)

a.非常同意 b.同意 c. 一般 d.不同意 e.非常不同意

2.您认为了解和学习艾滋病的前沿知识（或治疗进展）很重要。(P)

a.非常同意 b.同意 c. 一般 d.不同意 e.非常不同意

3.您会经常担心漏服HIV药物。(N)

a.非常同意 b.同意 c. 一般 d.不同意 e.非常不同意

4.您会因为需要携带药物或记住按时服药而感觉生活不便。(N)

a.非常同意 b.同意 c. 一般 d.不同意 e.非常不同意

5.您愿意接受长效疗法的治疗。 (P)

a.非常同意 b.同意 c. 一般 d.不同意 e.非常不同意

6.您认为长效疗法在HIV/AIDS治疗中的非常有优势。 (P)

a.非常同意 b.同意 c. 一般 d.不同意 e.非常不同意

7.您愿意推荐长效疗法给其他HIV感染者。 (P)

a.非常同意 b.同意 c. 一般 d.不同意 e.非常不同意

8.你认为长效疗法相较于传统每日口服药物可以减少治疗频率，提高生活质量。 (P)

a.非常同意 b.同意 c. 一般 d.不同意 e.非常不同意

9.你认为长效疗法相较于传统每日口服药物可以避免暴露隐私，对此抱有积极的态度。(P)

a.非常同意 b.同意 c. 一般 d.不同意 e.非常不同意

10.你认为长效疗法相较于传统每日口服药物可以减小因漏服药物导致的心理压力。(P)

a.非常同意 b.同意 c. 一般 d.不同意 e.非常不同意

11.你认为长效疗法相较于传统每日口服药物更加便利，比如出差或出游不用带药等。(P)

a.非常同意 b.同意 c. 一般 d.不同意 e.非常不同意

第四部分 HIV/AIDS患者对于长效艾滋病疗法的实践

1.您开始学习长效艾滋病疗法相关知识的情况：

a.经常 b.偶尔 c.从不

2.您知道吸烟的危害，如已吸烟，会尝试戒烟：

a.经常 b.偶尔 c.从不

3.您知道饮酒的危害，如已饮酒，会尝试戒酒：

a.经常 b.偶尔 c.从不

4.您会藏匿或伪装您的HIV药物，以避免被他人发现自己是HIV感染者？

a.经常 b.偶尔 c.从不

5.您会因为当前治疗方案的便利程度不佳而考虑更换治疗方案？

a.经常 b.偶尔 c.从不

6.您知道长效疗法需定期注射，您会遵医嘱按时注射（若采用长效疗法治疗）：

a.经常 b.偶尔 c.从不

7.您会按照医生要求做好注射治疗的自我护理（若采用长效疗法治疗）：

a.经常 b.偶尔 c.从不

8.您会主动进行相关检查（如血常规、肝功能、肾功能、CD4 细胞计数、病毒载量检测等）（若采用长效疗法治疗）：

a.经常 b.偶尔 c.从不

9.您会因为国内长效疗法刚起步而有所顾虑，比如疗效，副作用等

a.经常 b.偶尔 c.从不

再次感谢您参与填写我们的调查问卷，您回答所提供的信息对我们今后工作非常有价值！

Thank you for filling out our questionnaire！

若您对本次调查研究有任何意见与建议，我们将十分荣幸能够聆听您的声音。

意见与建议: ___________

为了本次问卷调研能够切实起到作用，推进今后回访工作的顺利开展，若您能够愿意留下联系方式，我们将不胜感激！

您的电话（选填）:_______________
