# Supplementary material for: Knowledge attitudes and practices towards long-acting antiretroviral therapy in HIV/AIDS patients
Source: Sci Rep. 2026 Mar 17;16:15840. doi: 10.1038/s41598-026-44035-0 (PMC13195075; doi:10.1038/s41598-026-44035-0)
Supplement: Supplementary file 3 — Supplementary Material 3 [file 41598_2026_44035_MOESM3_ESM.docx]

I acknowledge and agree that the collected data will be used for scientific research.

1. Yes, I agree to participate
2. No, I do not agree to participate

**Part 1: Basic Information**

1. Your gender:
   a. Male
   b. Female
2. Your age: ________
3. Your place of residence:
   a. Rural
   b. Urban
   c. Suburban
4. Your education level:
   a. Junior high school or below
   b. High school/technical secondary school
   c. Associate’s/bachelor’s degree
   d. Master’s degree or above
5. Your employment status:
   a. Employed
   b. Unemployed
   c. Retired or other situations
6. In the past year, your household’s monthly per capita income (including in-kind income, rental income, etc.): ______ yuan
   a. <2000
   b. 2000-5000
   c. 5000-10000
   d. 10000-20000
   e. >20000
7. Your marital status:
   a. Married
   b. Single
   c. Divorced, widowed, or other situations
8. How long has it been since your HIV diagnosis?
   a. Less than 1 year
   b. 1-3 years
   c. 3-5 years
   d. More than 5 years
9. Are you currently receiving treatment?
   a. Yes
   b. No
10. Has anyone around you experienced HIV infection?
    a. Yes
    b. No
11. Your height and weight:
    a. Height: ________ cm
    b. Weight: ________ kg
12. Have you heard of long-acting HIV therapy?
    a. Yes
    b. No
13. Have you ever had hypertension?
    a. Yes
    b. No
14. Have you ever had diabetes?
    a. Yes
    b. No
15. Do you smoke frequently?
    a. Yes
    b. No
16. Do you drink alcohol frequently?
    a. Yes
    b. No
17. If you choose long-acting therapy, the main reason is:
    a. Reduced dosing frequency, no need to think about taking medication often
    b. Fewer clinic visits
    c. Avoid side effects of oral medications
    d. Reduced risk of privacy exposure
    e. Avoid carrying large amounts of medication during business trips or travel
    f. Other (please specify): ________
18. If you do not choose long-acting therapy, the main reason is:
    a. Fear of injections
    b. High cost, financial pressure
    c. Inconvenience of regular visits to medical institutions for injections
    d. Concerns about side effects
    e. Worries about the efficacy of long-acting therapy
    f. Difficulty in adjusting treatment (e.g., dosage changes or medication switches)
    g. Other (please specify): ________

**Part 2 Knowledge of Long-Acting HIV Therapy among HIV/AIDS Patients**

For the following statements, please indicate your level of understanding:

1. AIDS (Acquired Immunodeficiency Syndrome) is a severe immune system disease caused by the Human Immunodeficiency Virus (HIV). HIV attacks the immune system, particularly CD4+ T cells, which are critical for coordinating the body’s defense against pathogens.

a. Very familiar

b. Heard of it

c. Unaware

1. HIV infection progresses through stages: *Acute infection*; *Asymptomatic/chronic infection*; *AIDS (late stage)*, where the immune system is severely damaged, leading to symptoms like persistent fever, weight loss, chronic diarrhea, fatigue, and swollen lymph nodes, etc.

a. Very familiar

b. Heard of it

c. Unaware

1. HIV treatment primarily relies on Antiretroviral Therapy (ART), which suppresses viral replication, restores immune function, reduces complications, and significantly prolongs life expectancy.

a. Very familiar

b. Heard of it

c. Unaware

1. Functional cure for HIV means that after stopping ART, the patient’s viral load remains undetectable, while CD4+ T cell counts and immune function stay normal.

a. Very familiar

b. Heard of it

c. Unaware

1. Beijing is the capital of China.

a. True

b. False

1. HIV replication can be suppressed using multiple antiretroviral drugs, including: *Nucleoside Reverse Transcriptase Inhibitors (NRTIs)*; *Non-Nucleoside Reverse Transcriptase Inhibitors (NNRTIs)*; *Protease Inhibitors (PIs)*; *Integrase Inhibitors (INSTIs)*; *Fusion Inhibitors (FIs); CCR5 Inhibitors, etc.*

a. Very familiar

b. Heard of it

c. Unaware

1. Common side effects of oral antiretroviral drugs may include: Nausea, vomiting, diarrhea; Headache, dizziness, depression, sleep disorders; Anemia, rash; Liver/kidney damage, lactic acidosis; Osteoporosis, increased lipids, weight gain, etc.

a. Very familiar

b. Heard of it

c. Unaware

1. Side effects of long-acting HIV drugs may include: Injection site pain; Gastrointestinal discomfort; Fever, fatigue; Liver function abnormalities; Anemia, headache, dizziness; Lipid/metabolic changes, blood sugar fluctuations, etc.

a. Very familiar

b. Heard of it

c. Unaware

1. Most antiretroviral drugs are oral (daily dosing), but some are injectable. Long-acting therapy mainly uses injectable combinations, eliminating the need for daily medication.

a. Very familiar

b. Heard of it

c. Unaware

1. China’s first approved long-acting HIV-1 regimen: *Cabotegravir injection + Rilpivirine injection* (administered every 2 months); *Cabotegravir tablets* can also be used with *Rilpivirine tablets* as an oral lead-in before injections or as a backup if injections are missed.

a. Very familiar

b. Heard of it

c. Unaware

1. Long-acting therapy is typically for patients with stable viral suppression and no drug resistance, meaning their viral load is undetectable or below a certain threshold.

a. Very familiar

b. Heard of it

c. Unaware

1. Research on long-acting HIV therapy aims to: Reduce dosing frequency; Decrease clinic visits; Prevent missed doses; Avoid oral drug side effects; Improve convenience and privacy.

a. Very familiar

b. Heard of it

c. Unaware

1. Despite advantages, long-acting therapy (e.g., Cabotegravir + Rilpivirine every 2 months) still requires professional injections, which may be burdensome, and side effects (e.g., GI discomfort, fever, fatigue, liver issues) remain possible.

a. Very familiar

b. Heard of it

c. Unaware

**Part 3 Attitudes of HIV/AIDS Patients toward Long-Acting HIV Therapy**

1. Currently, HIV cannot be cured and requires lifelong treatment, causing significant physical and psychological stress. *(Negative, N)*

a. Strongly agree

b. Agree

c. Neutral

d. Disagree

e. Strongly disagree

1. You believe it is important to learn about advancements in HIV treatment and research. *(Positive, P)*

a. Strongly agree

b. Agree

c. Neutral

d. Disagree

e. Strongly disagree

1. You often worry about missing doses of your HIV medication. *(Negative, N)*

a. Strongly agree

b. Agree

c. Neutral

d. Disagree

e. Strongly disagree

1. You find it inconvenient to carry medication or remember daily doses. *(Negative, N)*

a. Strongly agree

b. Agree

c. Neutral

d. Disagree

e. Strongly disagree

1. You are willing to try long-acting therapy for HIV treatment. *(Positive, P)*

a. Strongly agree

b. Agree

c. Neutral

d. Disagree

e. Strongly disagree

1. You believe long-acting therapy has significant advantages in HIV/AIDS treatment. *(Positive, P)*

a. Strongly agree

b. Agree

c. Neutral

d. Disagree

e. Strongly disagree

1. You would recommend long-acting therapy to other people living with HIV. *(Positive, P)*

a. Strongly agree

b. Agree

c. Neutral

d. Disagree

e. Strongly disagree

1. Compared to daily oral medication, you believe long-acting therapy reduces dosing frequency and improves quality of life. *(Positive, P)*

a. Strongly agree

b. Agree

c. Neutral

d. Disagree

e. Strongly disagree

1. Compared to daily oral medication, you believe long-acting therapy helps protect privacy, and you have a positive attitude toward this benefit. *(Positive, P)*

a. Strongly agree

b. Agree

c. Neutral

d. Disagree

e. Strongly disagree

1. Compared to daily oral medication, you believe long-acting therapy reduces psychological stress from missed doses. *(Positive, P)*

a. Strongly agree

b. Agree

c. Neutral

d. Disagree

e. Strongly disagree

1. Compared to daily oral medication, you believe long-acting therapy is more convenient (e.g., no need to carry medication while traveling). *(Positive, P)*

a. Strongly agree

b. Agree

c. Neutral

d. Disagree

e. Strongly disagree

**Part 4 Practices of HIV/AIDS Patients regarding Long-Acting HIV Therapy**

1. How often do you actively learn about long-acting HIV therapy?

a. Frequently

b. Occasionally

c. Never

1. If you smoke, how often do you try to quit, knowing the risks of smoking?

a. Frequently

b. Occasionally

c. Never

1. If you drink alcohol, how often do you try to reduce/quit, knowing the risks of alcohol?

a. Frequently

b. Occasionally

c. Never

1. Do you hide or disguise your HIV medication to avoid others knowing your status?

a. Frequently

b. Occasionally

c. Never

1. Would you consider switching treatments due to inconvenience with your current regimen?

a. Frequently

b. Occasionally

c. Never

1. If on long-acting therapy, would you adhere to scheduled injections as prescribed?

a. Frequently

b. Occasionally

c. Never

1. If on long-acting therapy, would you follow self-care guidelines for injection treatment?

a. Frequently

b. Occasionally

c. Never

1. If on long-acting therapy, would you proactively undergo required tests (e.g., blood tests, liver/kidney function, CD4 count, viral load)?

a. Frequently

b. Occasionally

c. Never

1. Do you have concerns about long-acting therapy due to its early-stage adoption in China (e.g., efficacy, side effects)?

a. Frequently

b. Occasionally

c. Never

Thank you once again for participating in our survey! The information you provided is extremely valuable for our future work.

Thank you for filling out our questionnaire！

If you have any feedback or suggestions regarding this study, we would be honored to hear your thoughts.

Comments/Suggestions: _________

To ensure this survey contributes meaningfully and facilitates follow-up research, we would be deeply grateful if you could optionally share your contact details.

Phone (optional): _______________
